# Prevalence, Characterization, and Pathogenicity of *Salmonella enterica* Subspecies *enterica* Serovar Derby from Yaks in the Aba Tibetan Autonomous Prefecture, China

**DOI:** 10.3390/ani11082397

**Published:** 2021-08-13

**Authors:** Xue Fu, Lan Feng, Linghan Kong, Chun Li, Xiaodong Zhao, Huade Li, Pengfei Cui, Wenjun Yan, Yaru Zhai, Lan Zhang, Hao Li, Hongning Wang, Xin Yang

**Affiliations:** 1 Key Laboratory of Bio-Resources and Eco-Environment, Ministry of Education, College of Life Science, Sichuan University, Chengdu 610064, China; 18482195563@163.com (X.F.); m15008448472@163.com (L.K.); cuipengfei@stu.scu.edu.cn (P.C.); yanwenjunibv@163.com (W.Y.); zhaiyr0806@163.com (Y.Z.); zl1007139545@163.com (L.Z.); lihaoibv@163.com (H.L.); whongning@163.com (H.W.); 2 Animal Disease Prevention and Food Safety Key Laboratory of Sichuan Province, Chengdu 610064, China; 3 Chengdu Centre for Disease Prevention and Control, Chengdu 610064, China; m18302822161@163.com; 4 Sichuan Animal Disease Control Center, Chengdu 610064, China; lich03@163.com; 5 Sichuan Longri Breeding Stock Farm, Hongyuan 624400, China; zxd77hzx@126.com; 6 Sichuan Academy of Grassland Sciences, Chengdu 610064, China; lihuade78@foxmail.com

**Keywords:** *Salmonella* Derby, yak, diarrhea, SNP analysis, virulence

## Abstract

**Simple Summary:**

*Salmonella* spp. is a very important pathogen in the livestock industry and public health, which poses a major threat to global public health. Yaks and their by-products have a significant economic status in the Qinghai–Tibetan Plateau. The aim of this study was to investigate the prevalence of salmonella in yak farms and to conduct a molecular characterization and tests on its pathogenicity in mice with the use of salmonella isolated from yaks with diarrhea as well as from drinking water samples. The prevalence of salmonella was 19.75% of 162 samples collected from yak farms, and all isolates were found to belong to the serovar of *Salmonella* Derby and ST40. All *Salmonella* Derby isolates from both fecal and drinking water samples from 13 farms were clonally related based on SNP alignment. *Salmonella* Derby was still detected positively in the feces of model mice on day 24 post-injection. This study reports the prevalence of *Salmonella* Derby in yaks with diarrhea and in their drinking water. In addition, the pathogenicity of the *S.* Derby in mice was investigated. Findings suggest that *Salmonella* Derby excreted by diarrheic yaks is a source of contamination for other yaks and the environment and is highly pathogenic to mice. Seeing that *Salmonella* Derby has become one of the most common *Salmonella* serovars, this situation gives rise to further risk from the potential spread of food-borne diseases.

**Abstract:**

*Salmonella enterica* subsp. *enterica* serovar Derby (*S.* Derby) is one of the numerous non-typhoidal *Salmonella* serovars and has been recognized as a food-borne pathogen. In 2019, outbreaks of salmonellosis were reported in 13 yak farms in the Aba Tibetan Autonomous Prefecture, China. A total of 32 salmonella strains were isolated from 162 fecal samples of yaks with diarrhea as well as from drinking water samples. The isolates were subjected to serovar identification, animal experiments, and whole-genome sequencing (WGS) analyses. The serovar of all the isolates was *S.* Derby, and the sequence types (STs) were ST40. The analysis of the differences of single-nucleotide polymorphisms (SNPs) showed that the salmonella strains isolated from 13 farms were clonally related. Animal experiments showed that the lethal dose (LD_50_) was 4.57 × 10^7^ CFU (colony-forming units); the shedding time of *S.* Derby in mice was 24 days; the bacterial loads in spleen were higher than those in other organs (ileum, liver, and cecum). Pathological analyses by hematoxylin and eosin (H&E) staining revealed obvious damage in the spleen, liver, and intestine. These results indicate that the *S.* Derby from yaks can cause infection in mice.

## 1. Introduction

*Salmonella* spp. is a very important pathogen in the livestock industry and public health, which poses a major threat to global public health [1]. It is considered to be a multi-host pathogen with long environmental persistence [2]. Since its first isolation, more than 2600 serovars of *Salmonella* spp. have been confirmed [3]. Except for some serovars that have host specificity such as *Salmonella (S.)* Gallinarum, most serovars have a wider host range, including chicken [4], pig [5], rabbit [6], sheep [7], and cattle [8], causing considerable economic losses to the livestock industry around the world. Two hundred eighty-seven serovars have been detected in China, which are dominated by *S.* Typhimurium [9]. *Salmonella* spp. is responsible for 70–80% of food-borne diseases in China and causes human epidemics, mainly through infection with salmonella-contaminated animals and plants [10].

*S.* Derby is one of the numerous non-typhoidal *Salmonella* serovars and has been recognized as a food-borne pathogen [11]. Since its first isolation in 1923, *S.* Derby has gradually spread worldwide [12,13,14,15]. The isolation rate of *S.* Derby in Europe has been increasing year by year; it is also the fourth most frequently isolated serovar in the non-human sectors in the United States [16,17]. In China, *S.* Derby has gradually become the main serovar isolated from slaughter pigs [18]. It is prevalent especially in pigs and pork products, with *S.* Derby from pigs being the main source of *Salmonella* spp. infection in humans [19]. Additionally, in a previous study performed in the United Kingdom between 2014 and 2015, the number of outbreaks increased more than fivefold among turkey flocks, indicating that this serovar is adapted to this particular host [20]. Altogether, *S.* Derby might represent a significant threat to human health, and the contributions of the pork and poultry sectors deserve to be further investigated.

Yaks (Bos grunniens) are mammals that are adapted to living in regions of high altitude in China, Russia, Nepal, and Mongolia [21,22]. The total yak population is estimated to be 14.2 million, with approximately 95% in China. Almost 1.52 million yaks live in the Aba Tibetan Autonomous Prefecture, which is located on the southeastern edge of the Qinghai–Tibetan Plateau. Yaks are essential animals for Tibetans and other nomadic pastoralists in high-altitude environments owing to their provision of the basic necessities (such as milk, meat, transportation, hides for tented accommodation, and dung for fuel) [22], and could thus cause the transmission of salmonellosis to humans through the food products they supply. However, the interspecies transmission of pathogens to yaks has significantly increased due to the complicated environment at high altitudes, where different species of animals share the same ecological resources [23]. Since the first report of salmonellosis in yaks in 1958, salmonella has been isolated and identified in yaks in the plateau area. The number of reports of yak salmonellosis in pastoral areas such as Qinghai, Gansu, Tibet, and Sichuan (Ganzi, Aba) has increased in recent years, suggesting that salmonellosis has become more prevalent among yaks. The *Salmonella* spp. serovars that infect yak are mainly *S.* Newport, *S.* Dublin, *S.* Saint-Paul, and *S.* Bovismorbificans, while infection with *S.* Derby has rarely been reported.

The isolation of *S.* Derby from feces of yak diarrhea in a Tibetan prefecture has already been reported in our previous case report in 2017 [24], which had few reports of infection with this serovar. We speculated that it might have been associated with yak diarrhea, but the molecular characteristics and prevalence of this serovar were unclear. In July and August of 2019, an outbreak of salmonellosis in yaks occurred. This study aimed to conduct a comprehensive microbiological investigation of *S.* Derby isolates recovered from clinical infection in yaks, including serovar identification, whole-genome sequencing (WGS) analyses, and its pathogenicity in mice.

## 2. Materials and Methods

### 2.1. Sample Collection

**Figure 1:**
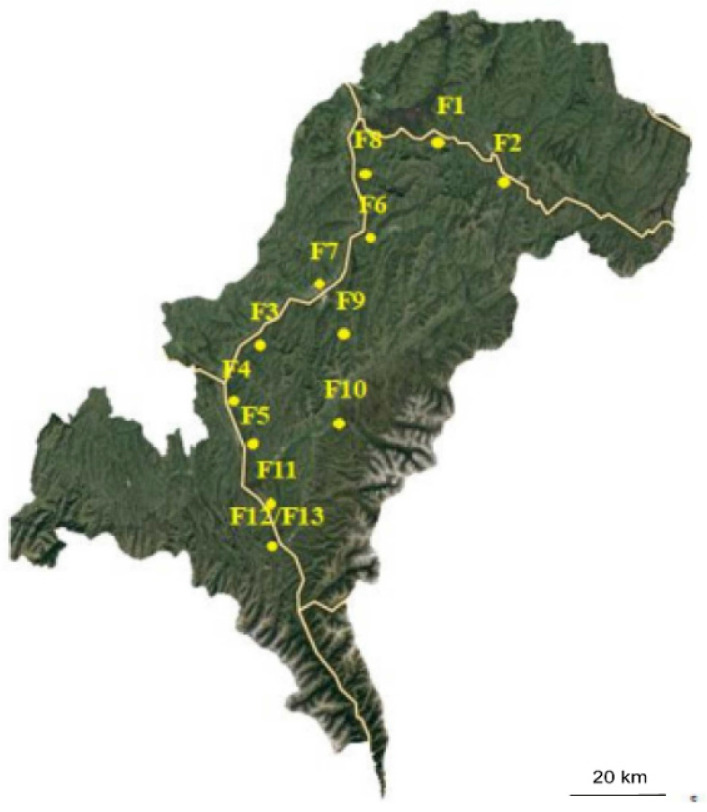
Approximate location of the isolates in the Aba Tibetan Autonomous Prefecture, China. The yellow line represents the river. F1–F13 are abbreviations for Farms 1–13. The latitude and longitude of farms 12 and 13 were the same.

In this study, 13 private yak farms (hereinafter referred to as F1–F13) located in the Aba Tibetan Autonomous Prefecture, with dispersed distribution, were selected; the locations of these farms are shown in Figure 1. The size of the farms varied between 300 yaks and 50 yaks; the yaks were free-ranged in their respective areas. We selected yaks with diarrhea symptoms and the number of samples collected in each field is shown in the Supplementary Table S1. The difference in sample sizes was due to the different number of diarrheic yaks at the sampling site. No mortalities were found at the time of sample collection. The average age of animals with diarrhea ranged from one month to three months. The onset of the disease was consistent with the typical clinical signs of diarrhea, i.e., obvious lack of spirit, low appetite, dry nasal mucosa, obvious dysentery. Fecal samples were mostly yellow-green and white; bloody fecal samples mostly watery, occasionally mucous and jelly-like, and accompanied by bad odor.

All samples were collected approximately between 5:00 a.m. and 6:00 a.m. (UTC+8) each day when yaks had just been released outdoors and produced fresh feces. The fecal samples were collected using sterilized cotton swabs and placed in aseptic tubes containing 5 mL of phosphate-buffered saline (PBS, HyClone, Shanghai, China), and drinking-water samples were taken from the river with sterile glass bottles. All samples were packed in sterile plastic bags, transported to the laboratory under cooled conditions, and processed the same day. A total of 162 samples (136 diarrhea fecal samples, 26 drinking water samples) were collected from yaks in 13 farms between July and August in 2019.

### 2.2. Salmonella Isolation

The fecal samples were pre-enriched with buffered peptone water (BPW, Beijing Bridge Technology, Beijing, China, incubated at 37 °C for 18 h; speed of 180 rpm), then selected and enriched with Rappaport’s 119 Broth (MM, Beijing Bridge Technology, Beijing, China, incubated at 42 °C for 24 h; speed of 180 rpm), and then isolated on selective xylose lysine tergitol-4 (XLT4) agar (Beijing Bridge Technology, Beijing, China, incubated at 37 °C for 24 h) for salmonella identification. Salmonella detection in water samples was performed with the use of filtration, concentration, pre-enrichment, selective enrichment, isolation by solid selective agar, and detection by the polymerase chain reaction (PCR) method [25]. In short, 500 mL of water per sample was filtrated using membranes with a pore size of 0.22 μm (Beyotime, Shanghai, China). The, membranes were then cut into pieces and placed in 100 mL of PBS. The solution was transferred into a 50 mL conical centrifuge tube and centrifuged (2500× *g*, 30 min). After aspirating the top solution, the remaining concentrated eluate was at 5 mL; the three experimental processes were subsequently conducted. A total of 1 mL of concentrate was added to 5 mL BPW and incubated for 16 h at 37 °C, with shaking. Then, 1 mL of the enriched culture was transferred to 10 mL of MM, after which triplicate parallel experiments were set up. These broths were streaked onto selective XLT4 agar and incubated at 42 °C for 24 h. According to the Kauffmann–White scheme [26],the isolated salmonella were serotyped using a microtiter agglutination test for O and H antigens following the manufacturer’s protocols (Diagnostic Sera for Salmonella, TianRun, Ningbo, China). The PCR products were then maintained at 4 °C and sequenced in both the forward and reverse directions by TsingKe (Chengdu, China). The sequences obtained were analyzed using BLAST [27].

Total DNA of the strain was extracted by boiling a single colony on the plate, as previously conducted [28]. Molecular identification of the isolated strains was achieved by PCR amplification of 16s rDNA, with the primers 27F (5′-AGAGTTTGATCMTGGCTCAG-3′) and 1492R (5′-TACGGYTACCTTGTTACGACTT-3′) [29]. Briefly, the cycling program comprised an initial denaturation of 5 min at 94 °C, followed by 30 cycles of amplification (denaturation at 94 °C for 1 min, annealing at 50 °C for 1 min, and extension at 72 °C for 1 min), with a final elongation at 72 °C for 10 min.

### 2.3. Whole Genome Sequencing (WGS) and Bioinformatics Analyses

A single colony of each isolate was inoculated into 3 mL of lysogeny broth (LB, Beijing Bridge Technology, Beijing, China) and incubated overnight at 37 °C, with shaking at 250 rpm. The samples were stored at −20 °C in a brain heart infusion broth (BHI, Beijing Bridge Technology, Beijing, China) containing 20% glycerol, for later use.

Then, genomic DNA was extracted using a TIANamp Bacteria DNA Kit (Tiangen, Beijing, China) following the manufacturer’s instructions. All salmonella isolates were sequenced by Beijing NovoGene Company using the Illumina Nova-seq PE150 technology sequencing platform (Illumina, San Diego, CA, USA) with the paired-end method. The data volume of each library was ≥90% of the target data volume to ensure that the total data quality met the required standard. Raw sequences were assembled through the Enterobase and finally submitted to the GenBank database. The Bioproject numbers of the 32 strains of salmonella in GenBank were PRJNA610941 and PRJNA640879.

Genome annotation was performed by RAST [30]. Multilocus sequence typing (MLST) based on seven housekeeping genes was performed, as previously described [31]. Alleles and sequence types were assigned according to the MLST scheme available online to determine the sequence type [32]. There were core genome MLST (cgMLST) based on 3002 loci and ribosomal MLST (rMLST) based on 53 genes encoding the bacterial ribosome protein subunits [33,34]. Single-nucleotide polymorphisms (SNPs) were determined using the CSI Phylogeny tool with its default settings [35]. Then, the evolutionary history was inferred by using the Maximum Likelihood method based on the Tamura-Nei model, and evolutionary analyses were subsequently conducted in MEGA7 [36,37]. An SNP analysis was performed with SD-13-18 (NCBI BioSample: SAMN15339256) as the reference genome, which was isolated in this study. Initial tree for the heuristic search was obtained automatically by applying the Neighbor-Join and BioNJ algorithms to a matrix of pairwise distances estimated using the Maximum Composite Likelihood (MCL) approach, and then selecting the topology with a superior log likelihood value. The Newick file was visualized using an online tool, the Interactive Tree Of Life (iTOL) v6 [38]. Five SNPs among isolates were considered to be clonally related and likely to have an epidemiological link [39,40]. The web server ResFinder version 4.1 was used for detecting acquired antimicrobial resistance genes [41]. The virulence of isolates was assessed through the detection of Salmonella Pathogenicity Islands (SPIs), while the virulence factors (VFs) were identified by using the SPI database, at the Center for Genomic Epidemiology (CGE) and with the virulence factor database (VFDB), respectively [42,43].

### 2.4. Animal Experiments

Thirty-six 4–6-week-old SPF female Kun Ming (KM) mice (weight 18–20 g) were purchased from the Animal Experimental Center of Sichuan University, China. All mice were housed in plastic cages and kept in an isolated biohazard cabinet for approximately 1 week of acclimatization. All mice had free access to the basal pellet diet and sterilized distilled water, and the cage bedding was changed twice weekly. The mice were treated based on the guidelines of the Committee for the Control and Supervision of Animal Experiments, and all protocols were approved by the Institutional Animal Ethics Committee (License: SYXK (Chuan) 2013-185) of Sichuan University.

The optical density at 600 nm (OD_600_) of the strain cultures was measured to identify the exponential growth phase and calculate the volume required to administer the correct dose of the strain by the corresponding linear regression analysis. All mice were randomly segregated into six groups (G1–G6; *n* = 6 mice per group). The inoculated salmonella strains were centrifuged, washed twice, and resuspended with sterile normal saline (Beyotime, Shanghai, China) to achieve an optical density (OD) value of 0.5. The mice in G1–G5 were intraperitoneally injected with the following doses of salmonella: 5 × 10^5^, 5 × 10^6^, 5 × 10^7^, 5 × 10^8^, and 5 × 10^9^ CFU (colony-forming units); the mice in the control group (G6) were injected with the same volume of sterile normal saline. The number of dead mice for each dose was recorded every day for 30 days, and then the lethal dose (LD_50_) at different time courses was determined using the Reed–Muench method [44].

From day 3 post-infection (pi) until the end of experiment at day 30 pi, feces were collected every 3 days to determine the duration of shedding and the bacterial loads of salmonella following inoculation. The enumeration analyses were performed, as previously described [45]. Briefly, fecal samples were diluted 1:10 in PBS, and serial dilution was performed in the LB until a dilution of 10^−5^ was obtained. Then, 100 μL of each of the dilutions was placed on three separate XLT4 plates. After incubation at 37 °C for 24 h, typical black colonies of salmonella were counted to enumerate salmonella in terms of CFU/g for each sample.

The spleen, liver, cecum, and ileum from euthanized mice in different groups were removed aseptically and homogenized in PBS to analyze *S.* Derby colonization. The bacterial loads were determined, as described above.

Hematoxylin and eosin (H&E) staining of the organs (intestine, liver, and spleen) were performed for histopathological examinations. Briefly, after collection during the necropsy, the samples were immediately fixed in 10% neutral buffered formalin, dehydrated in alcohol, embedded in paraffin, and then stained with H&E before being observed under a standard light microscope. The images of sections were obtained using a Ba200 digital micro camera (Motic China Group, Xiamen, China).

### 2.5. Statistical Analyses

Data were expressed as mean ± standard deviation and analyzed using GraphPad Prism Version 8.0.1 (GraphPad software, Inc., San Diego, CA, USA). The normality of the data (i.e., pairwise SNP differences) was checked using the Shapiro test, with R from the pairwise matrix generated by the CSI Phylogeny tool described above [46]. For all tests, *p* < 0.05 was used to indicate significant differences between the groups. Organ pathology was evaluated using a histopathological scoring scheme, as previously described [47].

## 3. Results

### 3.1. Prevalence and Serotyping of Salmonella

In this study, 32 salmonella isolates were obtained from 162 samples, with 28 (20.59%, 28/136) from fecal samples, 4 (17.40%, 4/26) from drinking water samples. Isolates were named using the format SD-F-N (S: salmonella; D: Derby; F: farm number; N: sample number; for instance, the twelfth sample collected from Farm 12 was named SD-12-12). The prevalence of 32 *S.* Derby strains is as follows: F1(1) (F1(1) indicates that there was one *S.* Derby isolated in farm 1), F2(2), F3(1), F6(2), F8(3), F9(1), F11(1), F12(7), and F13(12). The serovar of all isolates was *S.* Derby.

### 3.2. Molecular Typing of S. Derby and Whole Genome Sequencing (WGS) Anaylses

**Figure 2:**
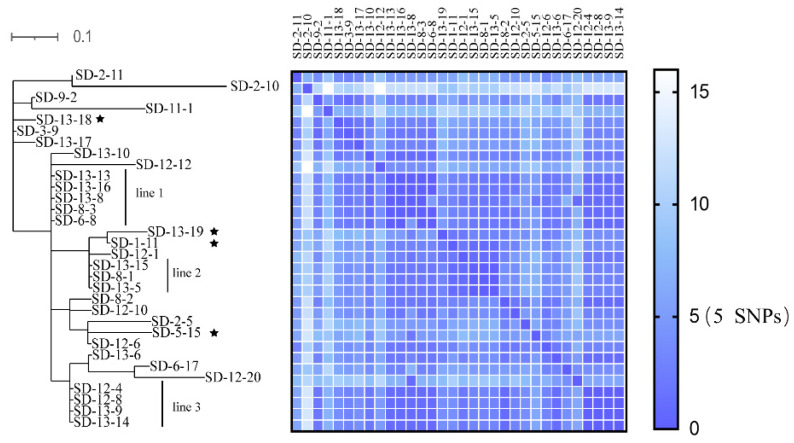
Molecular Phylogenetic analysis by Maximum Likelihood method. Phylogenies were inferred from Single-nucleotide polymorphisms (SNPs) and pairwise SNP distances (0–16 SNPs) are shown using a heat map. The tree is drawn to scale (0.1), with branch lengths measured by the number of substitutions per site. The analysis involved 32 nucleotide sequences. All positions containing gaps and missing data were eliminated. There was a total of 27 positions in the final dataset. SNPs between strains in different lines (line 1–3) were 0. Colors in the heat maps indicate the numbers of pairwise SNP distances between isolates, with blue being the lowest and white being the largest. The star markers indicate that the samples were isolated from drinking water.

All 32 *S.* Derby strains belonged to ST40 and rST28544, and further divided into 3 different cgSTs, mainly cgST198192. Only two strains were cgST198267 (SD-12-10) and cgST198266 (SD-13-17). The distances between each strain was 0–2 in the term of cgSTs. The phylogenetic tree based on the SNPs is shown in Figure 2; a total of 27 SNPs were identified. The SNPs among strains shown in Figure 2 as grouped in line 1, line 2, and line 3 were 0, indicating that strains from distinct farms were clonally related. Pairwise SNP distance between some strains from different farms were within 5 (shown in Figure 2). Whole genomic analysis of *S.* Derby carried multiple virulence genes, including genes encoding Type III secretion system (T3SS) effectors, adherence (shown in the Supplementary Table S2). In addition, other bioinformation analyses are shown in Supplementary Table S2.

### 3.3. Virulence of S. Derby in Mice

#### 3.3.1. LD_50_ Calculation

**Figure 3:**
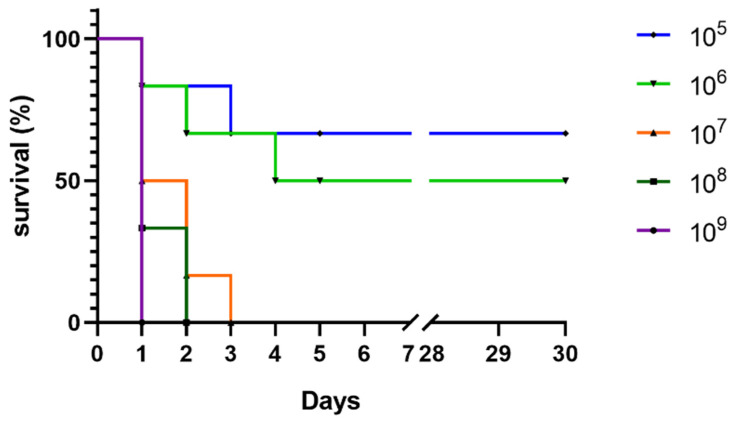
Female Kun Ming (KM) mice were challenged intraperitoneally with 5 × 10^5^–5 × 10^9^ CFU (colony-forming units) of bacteria. Mice survival 30 days after infection is shown; the mortality rate of the strain used was at 50% during the experimental period.

The survival curves of the mice challenged with *S.* Derby are shown in Figure 3. All mice in groups G1–G4 (5 × 10^5^–5 × 10^8^ CFU) showed diarrheal symptoms, which gradually decreased in the experimental process; mice in the 5 × 10^9^ group were dead on day 1 pi. The LD_50_ was measured as 4.57 × 10^7^ CFU.

#### 3.3.2. Fecal Shedding and Bacterial Loads in Organs

**Figure 4:**
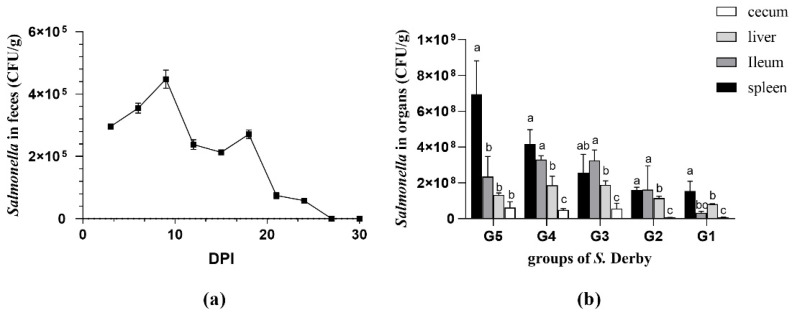
Fecal shedding and organ bacterial loads of the *Salmonella* (*S.)* Derby strain in the mouse model. (**a**) Fecal pellets were collected from the KM mice at the indicated time points for 30 days (or until the animal was sacrificed). The shedding of *S.* Derby was at about 24 days. Dots represent mean ± SD (Standard deviation) CFU (colony-forming units) counts in pellets of each mouse group; (**b**) Colonization in systemic and intestinal sites. Bacterial load in each mouse is represented as CFU/g by the individual column in the spleen (black columns), liver (light gray columns), ileum (gray columns), and cecum (white columns) at different doses. Group1-Group5 (G1–G5): The mice in G1–G5 were intraperitoneally injected with the following doses of salmonella: 5 × 10^5^, 5 × 10^6^, 5 × 10^7^, 5 × 10^8^, and 5 × 10^9^ CFU. Letters indicate significant differences between organs in each group after multiple comparisons.

Fecal shedding was monitored for 30 days. In *S.* Derby-infected mice, salmonella was detected from day 3 pi to day 24 pi, ranging from 5.8 × 10^4^ to 4.48 × 10^5^ CFU/g of feces (Figure 4). Feces of all animals in the control group remained culture-negative during the entire experiment.

**Figure 5:**
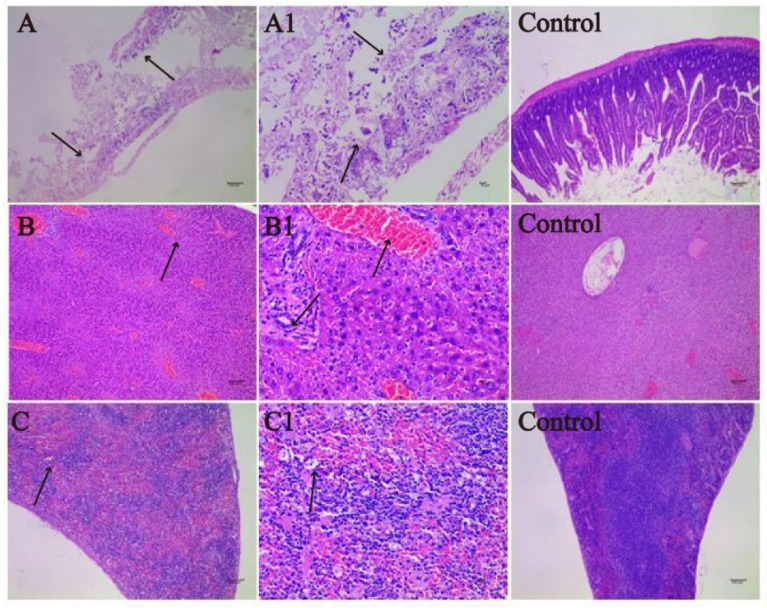
Histological lesions in the spleen, liver, and intestines of KM mice. (**A**) (100 × intestine)/(**A1**) (400 × intestine): the structure of intestines was destroyed, pointed to by the arrows in (**A**)/(**A1**); (**B**) (100 × liver)/(**B1**) (400 × liver): heterogeneous cytoplasmic staining results in the empty reticular structure, pointed to by the arrows in (**B**)/(**B1**); (**C**) (100 × spleen)/(**C1**) (400 × spleen): nucleolysis was observed, pointed to by the arrows in (**C**)/(**C1**). Scale bar: 100 μm ((**A**–**C**) and the Control group); 10 μm (**A1**–**C1**).

The results of salmonella colonization in each organ of the mice were ultimately concentrated at 10^7^–10^8^ CFU/g. Organs of all animals in the control group were culture-negative at day 30 pi. The colonization of *S.* Derby in four organs (spleen, ileum, liver, and cecum) had significant difference in all groups (*p* < 0.05; shown in Figure 5). *S.* Derby loads in the spleen were obviously higher than salmonella colonization in the cecum, followed by the liver in G5–G1. The cecum had the lowest concentration.

#### 3.3.3. Histopathological Analyzation

The H&E-stained sections of the spleen, liver, and intestines were subjected to histopathological analyzation. As shown in Figure 5A,A1, *S.* Derby caused intestinal damage. Severe focal necrosis was observed in the intestinal mucosa, with cell breakdown and disintegration, and some inflammatory cell infiltration (mainly lymphocytes) in the necrotic area. Intestinal gland atrophy was also observed to some extent.

In Figure 5B,B1, liver damage was observed, consisting of vacuolar degeneration of some hepatocytes around the central vein and portal canal area. The cytoplasm of hepatocytes was unevenly reticulated and dissolved to form vacuoles of different sizes. A few inflammatory cells, mainly lymphocytes and a few neutrophils, were scattered in the sinusoids of the liver. The pathological changes in the spleen (shown in Figure 5C,C1) included some lymphocyte infiltration of the white pulp splenic corpuscle, with punctate necrosis and a sparse arrangement of cells. The nuclei of necrotic cells were lost, and the red pulp of the spleen was slightly congested with increased erythrocyte infiltration.

## 4. Discussion

*Salmonella* spp. is increasingly present in cattle around the world, in those bred for both meat and milk production [48,49,50,51]. Guizelini et al. and Salaheen et al. found that most serovars of *Salmonella* spp. can infect and cause disease in cattle, leading to direct economic losses in the dairy industry associated with mortality or body weight loss, in addition to indirect losses caused by reduced feed conversion or medical care costs [52,53,54]. More than 90% of yaks in the world live in China and provide local residents with meat, dairy products, transportation, and fuel, which are vital to the meat and dairy production system in high-altitude regions [55]. *S.* Derby is one of the numerous non-typhoidal *Salmonella* serovars well-adapted to pig and turkey, and is also pathogenic to humans [56,57,58]. Since information about the *S.* Derby serovar isolated from yaks is still scarce, the aim of this study was to clinically investigate an outbreak of salmonellosis among yaks in several farms. We provided a comprehensive analysis of the molecular typing, WGS analysis, and the virulence of *S.* Derby isolated from yaks. This study reports the prevalence of *S.* Derby in yaks with diarrhea and in their drinking water. Although the clinical signs and contemporaneous occurrence of the outbreaks in all 13 farms suggested a diagnosis of *Salmonella* spp. infection, *S.* Derby was not isolated from yak feces taken from five farms (F1, F4, F5, F7, and F10). We speculated that the harsh and complex environment in the plateau area such as low temperature might have caused the yak diarrhea, which is a reminder that many more factors should be considered in future investigations.

Host adaptation is the ability of a serovar to circulate and persist in the population of a particular host species and may also be associated with distinct outcomes of infection. A previous study found that the STs of *S.* Derby corresponded to different hosts [57]. The most prevalent sequence type (ST) of *S.* Derby is ST40, which is considered to be well-adapted to swine and turkey [18,58]. A total of 2682 records of *S.* Derby ST40 from more than 20 countries have been uploaded to the EnteroBase Salmonella database (accessed 1 April 2021), indicating the international spread of ST40. In this study, there was no significant difference between the isolates at the genetic level. The 32 strains of *S.* Derby were all ST40, while ST40 was rarely found in yaks. In a previous study, the SPI-23 was only present in the swine-adapted lineages ST40 and ST39 [59,60]. Hayward et al. hypothesized that the host adaption of *S.* Derby might be distinguished by the presence and absence of SPI-23. In our study, we found that all 32 strains of *S.* Derby lacked SPI-23, which may provide further evidence for the hypothesis. Further investigation and characterization are required to verify the relationship between SPIs and *S.* Derby in host adaption. 

All *S.* Derby isolates from both fecal and drinking water samples from 13 farms were clonally related based on SNP alignment (Figure 2). The SNP differences between water samples (SD-1-11, SD-5-15, SD-13-18, and SD-13-19) were between four and five; the SPN differences between isolates from some farms were within five. The 13 farms surveyed were geographically distant from each other (Figure 1); the herders of each farm also seldom communicated and gathered. There was no transportation of yaks between each of the farms, and the yaks from each farm fed on the grass from their own grazing area throughout the year. Therefore, the possibility of salmonella transmission due to yak transfer is low. Some of the fecal samples, especially diarrhea fecal samples that were not easy to pick up, would flow into the river, which ran through local farms. The yaks in the surveyed farms used the river as a source of drinking water. Therefore, this may have infected the yaks as salmonella could survive in the water environment for a long time. The results may thus establish the hypothesis that there might be a clonal transmission between farms and a cross-contamination of salmonella.

We investigated the effect of infection caused by *S.* Derby in KM mice to better understand the progression of salmonellosis induced by *S.* Derby. In the pathogenicity study, the LD_50_ value of the original yak *S.* Derby was 4.57 × 10^7^ CFU, which indicated less pathogenicity than other *Salmonella* serovars and was consistent with previous studies [61,62,63]. Shedding of the *S.* Derby strain used was high at three days post-injection (DPI), and the maximum amount of salmonella excreted was observed at nine DPI; the *S.* Derby was detected positively at 24 DPI, at 2.15 × 10^6^ CFU/g of feces in mice, which showed that the bacterial shedding of feces was prolonged. Collectively, we established a KM mouse model to study infection by the *S.* Derby isolate and conducted its LD_50_, duration, and bacterial loads in organs. The findings of the present study demonstrated that *S.* Derby isolated from yak feces and their drinking water could successfully infect KM mice, leading to obvious clinical symptoms at high infectious doses. *S.* Derby also induced pathological damages to different organs in KM mice. The mice model for *S.* Derby infection established in this study provides an experimental animal model for the study of *S.* Derby infection and pathogenesis, and lays a foundation for further research. In the future, we intend to return to conduct relevant research on the yaks in order to provide more data for local salmonella control.

## 5. Conclusions

*Salmonella* spp. is a significant pathogen in food-producing animals, which are the primary source of salmonellosis [64]. Once introduced into the population, this can have widespread and serious consequences [65]. Thirteen geographically distinct farms were investigated for an outbreak of salmonellosis in yaks, with 32 *S.* Derby strains being isolated from 162 samples of feces and drinking water. These isolates were all ST40, which was considered to be well-adapted to swine and turkey, and all of them lacked the SPI-23. Otherwise, the phylogenetic tree and WGS analyses showed that these isolated strains were clonally related, indicating the clonal spread of the strains. Infection of yaks with *S.* Derby could facilitate the dissemination of salmonella into the food chain and its eventual transmission to humans. Furthermore, our study proved that *S.* Derby could induce pathological damages in KM mice. With the intrusive and damaging effects in KM mice, these results thus lay the basis for further studies on the virulence of *S.* Derby in yaks and humans alike. The present study also demonstrated the prevalence of *S.* Derby in yaks with diarrhea and in their drinking water, as well as its pathogenicity in mice. This study adds to our understanding of the *S.* Derby serovar isolated from yaks. Seeing that *S.* Derby has become one of the most common *Salmonella* serovars, this situation gives rise to further risk from the potential spread of food-borne diseases.

## Data Availability

The Bioproject numbers of the 32 strains of *Salmonella* in GenBank were PRJNA610941 and PRJNA640879.

## Supplementary Materials

The following are available online at https://www.mdpi.com/article/10.3390/ani11082397/s1, Table S1: Prevalence of Salmonella in yaks with diarrhea in Hongyuan, Tibetan Qiang Autonomous Prefecture of Ngawa, China, Table S2: Overview of complete genome sequence information of 32 salmonella strains.
