# Supplementary material for: Prevalence, Characterization, and Pathogenicity of Salmonella enterica Subspecies enterica Serovar Derby from Yaks in the Aba Tibetan Autonomous Prefecture, China
Source: Animals (Basel). 2021 Aug 13;11(8):2397. doi: 10.3390/ani11082397 (PMC8388676; doi:10.3390/ani11082397)
Supplement: Supplementary file 1 [file animals-11-02397-s001.zip › Supplementary table 2.pdf]

Supplementary Table S1 Prevalence of *Salmonella* in yaks with diarrhea in Hongyuan, Tibetan Qiang Autonomous Prefecture of Ngawa, China.

Samples	F1	F2	F3	F4	F5	F6	F7	F8	F9	F10	F11	F12	F13	Total
Fecal	0/10	3/15	1/16	0/6	0/14	2/24	0/8	3/3	1/2	0/1	1/2	8/18	9/17	28/136
Water	1/2	0/2	0/2	0/2	1/2	0/2	0/2	0/2	0/2	0/2	0/2	0/2	2/2	4/26
Total	12	17	18	8	16	26	10	5	4	3	4	20	19	162
Positive samples (%)	8.33%	17.65%	5.56%	0	6.25%	7.69%	0	60.00%	25.00%	0	25.00%	40.00%	57.89%	19.75%
